# Intranasal breast milk for premature infants with severe intraventricular hemorrhage—an observation

**DOI:** 10.1007/s00431-018-3279-7

**Published:** 2018-11-01

**Authors:** Titus Keller, Friederike Körber, André Oberthuer, Leonie Schafmeyer, Katrin Mehler, Kathrin Kuhr, Angela Kribs

**Affiliations:** 1 Division of Neonatology, Children’s Hospital, University of Cologne, Cologne, Germany; 2 Department of Radiology, University of Cologne, Cologne, Germany; 3 Institute of Medical Statistics, Informatics and Epidemiology, University of Cologne, Cologne, Germany

**Keywords:** Nasal application, Breast milk, Neonatal brain injury, Stem cells, Neurotrophins

## Abstract

For nasal application of neurotrophins and mesenchymal stem cells, successful delivery to the brain and therapeutic effects are known from experimental data in animals. Human breast milk contains neurotrophins and stem cells, but gavage tube feeding in preterm infants bypasses the naso-oropharynx. This is a first exploration on additional nasal breast milk and neuromorphological outcome after severe neonatal brain injury. We present a retrospective summary of 31 very low birth weight preterm infants with intraventricular hemorrhage °3/4 from one third-level neonatal center. All were breast milk fed. Sixteen infants additionally received nasal drops of fresh breast milk daily with informed parental consent for at least 28 days. Cerebral ultrasound courses were reviewed by a pediatric radiologist blinded to the intervention. The main outcome measure was severity of porencephalic defects before discharge. Clinical covariates were comparable in both groups. With nasal breast milk, a trend to a lower incidence for severe porencephalic defects (21% vs. 58%) was detected. Incidences were lower for progressive ventricular dilatation (71% vs. 91%) and surgery for posthemorrhagic hydrocephalus (50% vs. 67%).

*Conclusion*: The hypothesis is generated that early intranasal application of breast milk could have a beneficial effect on neurodevelopment in preterm infants. Controlled investigation is needed.

**Table:** 

**What is Known:** • *Successful delivery to the brain and therapeutic effects are known for nasal application of neurotrophins and mesenchymal stem cells from experimental data in animal studies*. • *Human breast milk contains neurotrophins and stem cells, but gavage tube feeding in preterm infants bypasses the naso-oropharynx*.	
**What is New:** • *This is the first report on additional nasal breast milk application in very low birth weight preterm infants with severe brain injury observing a trend for less severe porencephalic defects.* • *The hypothesis is generated that nasal breast milk might exert neuroprotective effects in preterm infants*.

**Electronic supplementary material:**

The online version of this article (10.1007/s00431-018-3279-7) contains supplementary material, which is available to authorized users.

## Introduction

Preterm infants < 1500 g (very low birth weight, VLBW) are a patient cohort with significant morbidity and mortality. Severe intraventricular hemorrhage (IVH) remains an important complication with limited therapeutic options [32]. Erythropoetin is one therapeutic approach with reported positive effects on neurologic outcome [16]. Furthermore, data from animal studies support the hypothesis that intranasal application of neuroprotective substances, e.g., epidermal growth factor (EGF), may constitute a future therapeutic approach [29]. However, there is a lack of data from human studies with intranasal treatment approaches.

It is known that breastfeeding is associated with improved performance in intelligence tests years later [14, 18]. Observational studies indicate a positive effect of breast milk (BM) fed via gavage tube on neurologic long-term outcome of preterm infants [15, 22, 31]. However, the mechanisms of the beneficial effects on neurodevelopment are not well understood. Furthermore, the impact of breastfeeding on IVH and periventricular leukomalacia is not well investigated to date. It is well-known that breast milk is rich in growth factors, neurotrophins, and immune cells [2, 13]. The exposure of the naso-oropharyngeal mucosa of healthy newborns to their own mother’s milk occurs physiologically during breastfeeding. But in VLBW infants on a neonatal intensive care unit (NICU), this contact is usually bypassed by feeding via gavage tube, a small tube that is placed up the nose into the stomach [11]. So far, the role of nasal breast milk exposure for effects on the central nervous system has never been investigated.

At our tertiary neonatal center, more than 85% VLBW infants are fed with breast milk. We offered a few drops of additional breast milk intranasally to preterm VLBW infants after diagnosis of severe intraventricular hemorrhage grade 3/4 in the form of a compassionate use following parental informed consent. A positive outcome in the cerebral ultrasound courses of these patients was observed. In this paper, we therefore present a retrospective summary of the clinical courses and longitudinal ultrasound findings of this small series of VLBW infants.

Our aim is to explore the neuromodulatory potential of intranasal breast milk after severe neonatal brain injury in preterm infants with very low birth weight on neuromorphological outcome.

## Materials and methods

### Design

We present a retrospective case-control study of cerebral ultrasound courses after severe intraventricular hemorrhage in very low birth weight preterm infants to examine the exposure to intranasal breast milk. The use of intranasal breast milk was performed as compassionate use in a situation with limited therapeutic options with the idea to possibly enhance the beneficial effect of breast milk on neurodevelopment.

**Fig. 1:**
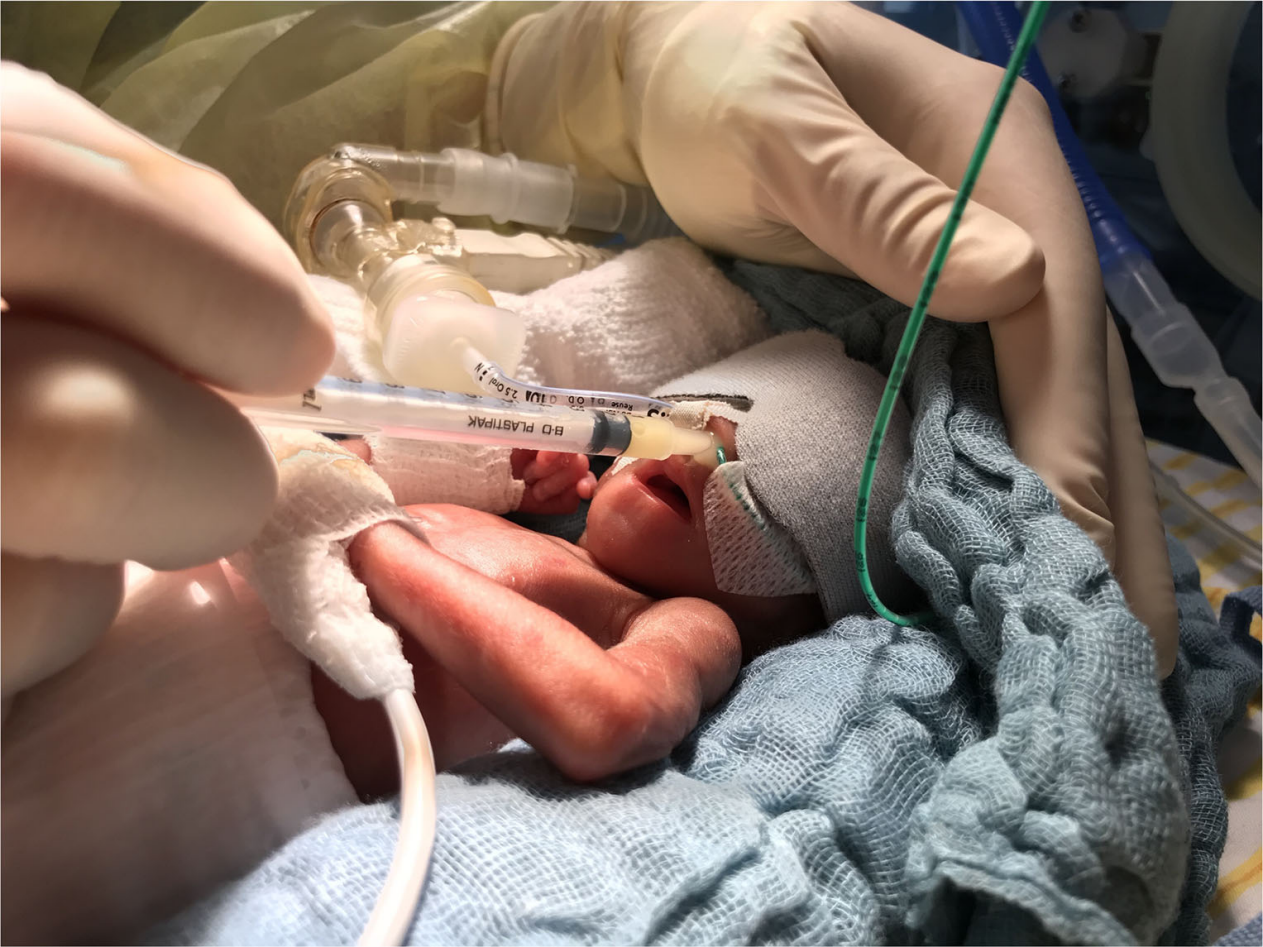
Intranasal breast milk application

Small syringes (1 ml) were used to administer 0.1 ml of breast milk per nostril (Fig. 1). Each neonate received 2 × 0.1 ml of his or her mother’s milk 3 to 8 times a day, so the daily volume of intranasally administered breast milk ranged between 0.6 and 1.6 ml. The breast milk was freshly expressed, which means the milk was used within 2 h after expression. The daily application started within the first 5 days of life and was continued for at least 28 days to a maximum of 105 days.

### Setting

All infants in this study were inborns at one third-level neonatal intensive care unit with 120 VLBW infants per year. In July 2012, one VLBW infant with a severe IVH started receiving regular drops of fresh milk from her mother intranasally out of compassionate use. The eutrophic female preterm infant of 26 weeks was born after preterm labor by spontaneous vaginal delivery within the complete amniotic sac (Apgar score 7/8/9, umbilical artery pH 7.25). Delivery room management followed the local standard protocol including prolonged placental transfusion, respiratory stabilization with continuous positive airway pressure, and less invasive surfactant application [23]. The infant had skin to skin contact with the mother in the delivery room for 1 h before transport to the NICU. No cardiorespiratory problems and no signs of sepsis under standard antibiotic regimen occurred. No transfusions were needed. Weaning from CPAP was possible on day 9 and from any nasal flow at day 40 with caffeine therapy for 68 days. Feeding with breast milk was started from the first day with small amounts of colostrum via nasogastric tube and some drops orally on a cotton bud.

On DOL 3, the cerebral ultrasound (cUS) showed a bilateral IVH right grade II and left grade III with intraparenchymal infarction on the left side. Explaining the diagnosis to the parents included explanation of possible consequences as well of the limited therapeutic options. Facing the question if any other measures beyond standard therapy could be taken to enhance any possible beneficial effect on brain development, the additional intranasal application of some drops of breast milk was proposed by the attending neonatologist. The idea was to possibly increase exposure of the brain to neurotrophins and stem cells in breast milk via the nasal route. The parents gladly consented to a compassionate use. Since the measure itself is common use for nasal congestion in neonates, there were no general safety concerns. To avoid loss of positive end-expiratory pressure during CPAP therapy, nose drops were given during the regular rounds of switching the nasal CPAP device. One drop of fresh milk from the baby’s own mother was administered per nostril three times daily and continued for 10 weeks until discharge. The IVH resolved over a period of 7 weeks, and the neurologic evaluation at discharge was good. The intriguing good course of this child contributed to the application of the same measure in 15 other VLBW with severe IVH between July 2012 and August 2014 with informed parental consent. Only one VLBW infant with a severe IVH in this time frame did not receive intranasal breast milk before the course of the first child was assessed. A major change in spatial terms and conditions of this NICU in 2015 excluded comparison to a more recent control group, which would have introduced major confounders. Therefore, the search for a control group of appropriate cases was extended for 2 years before the index patient.

### Sample

We included all VLBW infants with IVH grade 3/4 born at the same center from August 1, 2010 to August 31, 2014 who survived the first 3 days of life and received milk from their own mothers. From August 1, 2010 to August 31, 2014, 39 VLBW infants born at the same center developed an IVH grade 3/4 within the first 5 days of life. Three of these infants died within the first 3 days of life. Five of the 36 surviving infants did not receive breast milk for maternal reasons. Among the 31 breast milk-fed infants with IVH °3/4 that survived after the third day of life, 16 infants received additional intranasal breast milk while the other 15 did not. The intranasal application started within the first 5 days of life and was performed at least daily over the first 28 days of life and in most cases until discharge.

### Measurement

**Fig. 2:**
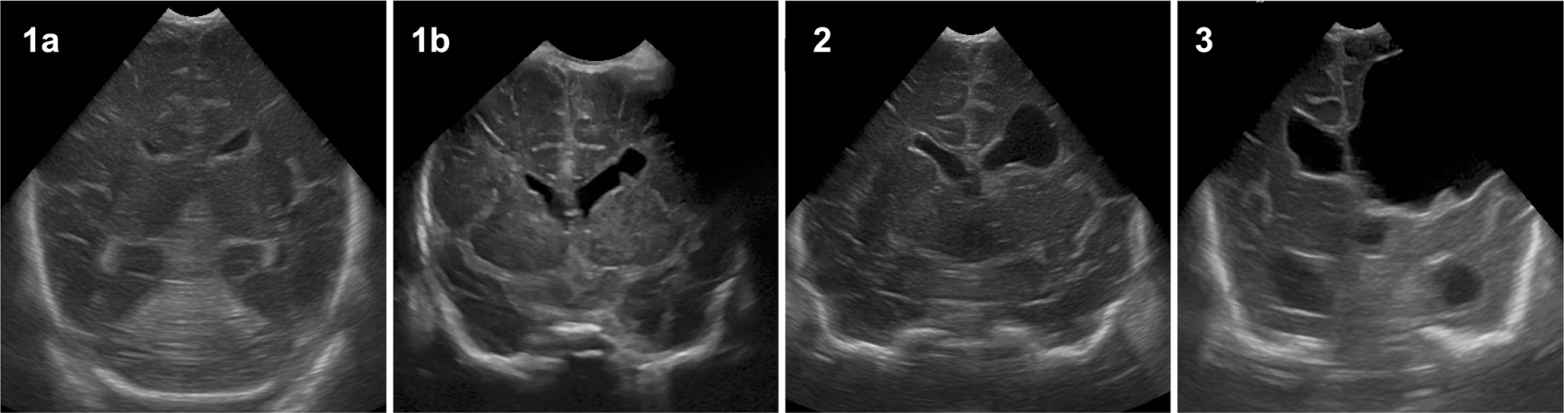
Classification of white matter damage by cystic lesion size at discharge: Exemplary coronal cerebral ultrasound slices of cystic white matter damage at time of discharge (postmenstrual age of 41 ± 5 weeks). **1a** Gliosis optionally with cystic defects without communication to the ventricles. **1b** Small cystic defect with communication to the ventricles. **2** Moderately sized cystic defect with communication to the ventricles. **3** Large cystic defect with communication to the ventricles

Standard cerebral ultrasound diagnostic was performed daily until day three, then weekly. After 4 weeks, ultrasound was performed every other week. For this analysis, the cerebral ultrasound images were evaluated by a pediatric radiologist blinded to the exposure to intranasal breast milk as well as date of scan. The initial parenchymal bleeding was classified according to the classification by Papile, for more precise description of the severe forms we defined the subgroups 3–4, 4+, and 4++ (definition is given in Table 2). This initial classification describes the finding before any inBM exposure. For evaluation of progressive ventricular dilatation, the definition of Murphy et al. was applied [24]. For classification of white matter damage (WMD) findings at discharge around 41 weeks of postmenstrual age, we defined four groups. The periventricular cysts were classified according to size grouped in small, moderate, and large cystic defects. Small cysts did not exceed diameter of the lateral ventricles. Moderate cysts did not exceed half the diameter of the hemisphere; large cysts did exceed half the diameter of the hemisphere. An additional group was added to the small-sized cysts, because here cysts with and without communication to the ventricles could be found, while there were no moderate or large cysts without communication to the lateral ventricles. Both hemispheres were evaluated separately. The severest finding was considered for analysis. White matter classification is presented with exemplary ultrasound images in Fig. 2.

The clinical history was reviewed from the patients’ charts. Written parental informed consent was ensured before publication of pictures. No ethical concerns were declared by the ethical board of the University of Cologne (No. 17-057).

### Data analysis

Statistical analyses were performed using IBM SPSS Statistics 23 (SPSS Inc., Chicago). Data were described using median (range), mean ± standard deviation, or frequencies and percentages. We used Mann-Whitney *U* tests for continuous variables and Fisher’s exact test for categorical variables to perform pairwise group comparisons. All reported *p* values are two-sided and *p* values < 0.05 were considered statistically significant. Due to the exploratory character of this study, we did not adjust for multiple testing.

## Results

Among 39 inborn VLWB infants that developed a severe IVH grade 3 or 4, three infants died within the first 3 days. Breast milk feeding was initiated in 31 survivors, and 16 of these received additional breast milk intranasally. Perinatal data arranged according to administration of intranasal breast milk of these infants is presented in Table 1. Within the first 2 weeks of life, three children died in the control group while two children died in the intervention group. There were no significant differences in all clinical covariates between control and intervention group.

**Table 1:** Clinical covariates

Clinical covariates	Infants, No. (%)			*p* value^a^
	No inBM	inBM	Total	
Total No.	15	16	31	
Birth weight mean ± SD, g	684 ± 218	770 ± 245	728 ± 232	0.308
GA mean ± SD, weeks	24.4 ± 1.6	24.9 ± 1.6	24.7 ± 1.6	0.444
Male sex	11 (73)	9 (56)	20 (65)	0.458
Multiples	6 (40)	7 (44)	13 (42)	1.000
Cesarean delivery	11 (73)	11 (69)	22 (71)	1.000
Apgar median (IQR)				
At 5 min	6 (4–7)	7 (6–7.75)	6 (5–7)	0.141
At 10 min	8 (6–8)	8 (7–8)	8 (7–8)	0.403
Survived until discharge	12 (80)	14 (88)	26 (84)	0.654
Mechanical ventilation mode				0.288
High-frequency oscillation	11 (35)	10 (32)	21 (68)	
Conventional	1 (3)	1 (3)	2 (6)	
Non-invasive ventilation only	0 (0)	3 (10)	3 (10)	
MV duration of survivors, median (IQR), days	17.5 (6.5–33.75)	10.0 (7.25–15.25)	12.0 (7–21.25)	0.252
CRIB score median (IQR)	11 (7–12.5)	9 (5–10)	10 (5–11)	0.140
FIP	0/12 (0)	3/14 (21)	3 (10)	0.225
Laser therapy/anti-VEGF for ROP	0/12 (0)	2/14 (14)	2 (6)	0.483
BPD at 36 weeks	6/12 (50)	6/14 (43)	12 (39)	1.000
Hospital stay of survivors, median (IQR), days	119 (80.75–148.5)	107.5 (82–124.25)	109 (82–137.25)	0.308

*inBM* intranasal breast milk, *CRIB* Clinical Risk Index for Babies, *MV* mechanical ventilation, *FIP* focal intestinal perforation, *ROP* retinopathy of prematurity, *BPD* bronchopulmonary dysplasia

^a^Mann-Whitney *U* test for continuous variables, Fisher’s exact test for categorical variables

The course of cerebral ultrasound findings is summarized in Table 2. Severity of IVH findings within the first days were similar in both groups. The incidence for the severest IVH findings were slightly higher in the inBM group (64%) than in the control group (58%) without statistical significance. Nevertheless, incidence for progressive ventricular dilatation was lower in the intranasal breast milk group than in the control group as well as incidence for surgery for posthemorrhagic hydrocephalus.

**Table 2:** Short-term outcome and course of cerebral ultrasound findings

Outcome		Infants, No./total No. (%)		*p* value^a^
		No inBM	inBM	
Death within first 2 weeks		3/15 (20)	2/16 (13)	
Survivors until discharge, *n*		12/15 (80)	14/16 (88)	0.654
Survived and needed no surgery for hydrocephalus		4/15 (27)	7/16 (44)	0.458
IVH grading among survivors, maximal finding initially				
3–4	Higher periventricular echolucency suspected for infarction	3	2	
4	Definitive periventricular infarction	2	3	
4+	Emphasized periventricular infarction	7	8	
4++	Extended periventricular infarction	0	1	
Severest findings (IVH 4+ and 4++)		7/12 (58)	9/14 (64)	1.000
Progressive ventricular dilatation		11/12 (91)	10/14 (71)	0.330
Surgery for posthemorrhagic hydrocephalus		8/12 (67)	7/14 (50)	0.453
White matter damage detectable by cUS, maximal finding at discharge				
0	no	4 (27)	5 (31)	1.000
1a	Gliosis optionally with small cystic defects without communication to the ventricles	1	2	
1b	Small cystic defect with communication to the ventricles	0	4	
2	Moderate cystic defect with communication to the ventricles	5	2	
3	Large cystic defect with communication to the ventricles	2	1	
Severest findings (WMD 2 and 3)		7/12 (58)	3/14 (21)	0.105

*inBM* intranasal breast milk, *cUS* cerebral Ultrasound, *WMD* white matter damage

^a^Fisher’s exact test

Severity of white matter damage was classified depending on size of cystic defects detected by ultrasound at discharge. An exemplary ultrasound course with images for 8 different time points of one patient from initial finding to finding at discharge is presented in Supplementary File 1 showing the resolve of an initial IVH 4+ to final WMD 1a. A short summary of six cases with intriguingly good courses with intranasal breast milk is highlighted in Supplementary File 2 showing the initial ultrasound finding and the last finding before discharge.

The severest findings in white matter damage in the form of moderate to large cystic defects with communication to the lateral ventricles were found 2.8 times more often in the control group than in the intervention group. Statistical significance by Fisher’s exact test was not found.

## Discussion

Therapeutic neuroprotective strategies for severe brain injury of preterm infants are not yet accessible for clinical use. Experimental data support high efficiency for nasal application of growth factors and stem cells for rescuing neonatal brain injury. Breast milk contains a multitude of both. Clinical studies indicate that breast milk feeding of preterm infants improves long-term neurocognitive outcome. The idea of enhancing a neuroprotective effect of breast milk by nasal application in addition to the standard way of gavage feeding in very low birth weight preterms led to a compassionate use in 16 infants with severe intraventricular hemorrhage °3/4. Periventricular cystic defects following severe IVH document destruction of white matter in form of colliquative necrosis and can be detected via cerebral ultrasound at discharge. This case-control analysis reveals a trend for a better resolve of severe IVH after the additional intranasal exposure to breast milk. Our observation demonstrates the feasibility of this measure and warrants discussion as a hypothesis in need to be tested.

The large randomized PROBIT Trial provided strong evidence that prolonged and exclusive breastfeeding improves children’s cognitive development [18]. Observational studies on preterm infants indicate that feeding with breast milk on the neonatal intensive care unit improves long-term neurocognitive outcome significantly [15, 28, 31]. Preterm infants at NICU are normally fed via gavage tube due to sucking weakness and in order to avoid aspiration, especially when in need of respiratory support. Here, the oropharynx is bypassed. Lee et al. noted in his RCT that additional oropharyngeal administration of colostrum significantly reduced incidence of clinical sepsis, inhibited secretion of pro-inflammatory cytokines, and increased levels of circulating immune-protective factors in extremely low birth weight infants [20]. This suggests that the oropharyngeal exposure with colostrum has more relevance than known before.

In the healthy newborn, the act of sucking-swallowing-breathing during breastfeeding allows fresh milk to get in contact with the naso-oropharynx. This can be observed when BM spontaneously drops out of the nose of the breastfeeding newborn. It occurs even more often in the immature swallowing coordination in preterms. Giving nose drops from neonates’ own mothers’ milk is a simple and common measure for treating their nasal congestion. Despite the close relationship of the nasal cavity to both the oropharynx and the nose-brain-access via the olfactory neural pathway, there have until now been no reports on nasopharyngeal exposure to breast milk with focus on effects on brain development.

The ongoing brain growth spurt of the preterm newborn presents an optimal time window for a continuous exposure to neurotrophic substances [6].

Two considerations add to the plausibility of a noteworthy hypothesis. First, human breast milk contains numerous neurotrophic factors such as EGF, brain-derived neurotrophic factor, glial-derived neurotrophic factor, nerve growth factor, insulin-like growth factor-1, and hepatic growth factor [17]. Neurotrophins are considered to potently support the development, growth and survival of neurons, eliciting biological effects at concentrations in the nanomolar range. EGF concentrations in milk are considerably higher than in maternal serum, tenfold higher in colostrum than in mature milk, and even higher in the milk of extreme preterms (around 160 ng/ml) compared to terms (80 ng/ml) [9, 26]. Breast milk contains lactoferrin in high concentrations. Neuroprotective effects of lactoferrin on the immature brain have been shown in rodent models of intrauterine growth restriction, cerebral hypoxia/ischemia and lipopolysaccharide-induced brain injury [12, 30]. Stem cells with multipotent and even pluripotent properties have been detected in human breast milk ranging between < 1 and 30% of total breast milk cells [2, 13, 25]. Breast milk therefore contains several factors with neuroprotective potential. Second, intranasal drug delivery is emerging as a reliable method to bypass the blood-brain-barrier and to deliver a wide range of therapeutic agents including growth factors and stem cells directly to the brain [21]. Scafidi applied EGF intranasally to a neonatal mouse model of very preterm brain injury and described enhanced generation of new oligodendrocytes and improvement in functional recovery [29]. Danielyan et al. showed for the first time the nasal delivery of mesenchymal stem cells to the naïve mouse brain and the route of their migration crossing the cribriform plate into the brain [5]. Donega et al. described the successful delivery and therapeutic effects of MSCs in a neonatal hypoxic-ischemic brain injury model using unilateral carotid ligature [7, 8]. Neuroprotective and regenerative effects have been demonstrated in neonatal animal models for intranasal neurotrophins and MSCs, such as stimulating oligodendroglia growth, neurite outgrowth, reduced lesion volume, and improved neurological outcome [7, 29].

Clinical trials have shown the successful use of intranasal insulin to safely improve memory and cognition in patients with mild cognitive impairment or Alzheimer’s disease without alteration in the blood levels of insulin or glucose [3, 27]. This demonstrates that intranasal insulin, a therapeutic growth factor, reaches the brain through nose-to-brain delivery without relying on the bloodstream, and it demonstrates an increased safety by avoiding unwanted systemic exposure. In this way, efficiency and safety have been demonstrated for intranasal delivery of growth factors in humans [4, 21].

The uniqueness of olfactory neurons exposed to the external environment serving as an entrance to the brain is not selective for neurotrophins or stem cells. Of course, pathogens such as the ameba *Naegleria fowleri* also use the nasal route to reach the brain [19]. Therefore, the olfactory route of neuroinvasion needs critical attention when intranasal delivery of the nonsterile breast milk is considered. In cases of maternal infection, the breast milk should not be used for nasal application without reasonable care, and microbiological testing for neurovirulent pathogens should be considered.

Data on the neuromodulatory effect of human milk after brain injury in preterms is still lacking. While efforts have been undertaken to research stem cells for regenerative therapy for neonatal brain injury, little is known about the physiological supply of the neonate with neurotrophins and stem cells, particularly for the vulnerable or impaired brain of preterms via breast milk, even though there is evidence for the beneficial effect of breast milk on neurodevelopment.

A randomized controlled trial (RCT) on breast milk feeding versus formula is not to be expected, because randomization of breast milk feeding is ethically not acceptable in view of the evidence for multiple benefits on infants’ health [1]. Here, the insights in the efficiency of the nose-brain-transport might introduce a unique opportunity to investigate the effect of breast milk itself on neurodevelopment in preterm infants in an RCT comparing additional nasal application versus nasal placebo in breast milk fed preterms.

To examine the effect of intranasal breast milk on neuronal/axonal damage, investigation by MRI with diffusion tensor imaging would be appropriate. Early assessment of general movements would help improve predictive value for long-term outcome [10]. Since a stimulating effect on the brain growth spurt in preterm and term neonates is to be expected independent of a previous brain injury, this also needs to be subject to further investigation. Systematic research on composition in view of neurotrophic factors and the functions of cells in breast milk of the most immature preterm infants could help identify candidates of outstanding relevance and help understand the importance of the composition.

### Limitations

This is a retrospective analysis so that confounders cannot be completely excluded. To exclude known confounders such as gestational age, infection, and mechanical ventilation, identical inclusion criteria were applied to all included infants.

The study fails to reach statistical significance. The number of patients per year even at a neonatal center with high annual numbers of preterm newborns is limited by the fortunately low incidence of severe intraventricular hemorrhage. Additionally, a profound change in spatial conditions at the same center after the period of observation would have introduced major confounders.

A morphological finding of brain injury is reported. Even though large cystic defects are predictive for cerebral palsy, a functional outcome would be of major interest. Neurodevelopmental outcome data were collected and could be accessed on demand but were not complete and difficult to compare for usage of different instruments (BSID II or III) at different time points and in part at different centers, which did not allow for a conclusive interpretation.

## Conclusion

A trend for less severe cystic defects after severe intraventricular hemorrhage in very low birth weight infants was observed for exposure to intranasal breast milk. In synopsis with the experimental data on successful delivery of neurotrophins and stem cells via the nasal route to the brain, together with the presence of both in breast milk, the hypothesis is generated that nasal exposure to fresh milk from neonates’ own mother may contribute to an enhanced delivery of neuromodulatory substances in breast milk to the neonatal brain.

The need for therapeutic options in severe neonatal brain injury and the available supply of a complex biocompatible liquid containing neurotrophins and stem cells warrants further controlled investigation.

## Electronic supplementary material

Supplemental File 1 Exemplary longitudinal cerebral ultrasound course of one infant at eight timepoints. Male infant of 22 weeks of gestational age with bilateral IVH 4+. At discharge bilateral small periventricular gliosis. (JPG 3496 kb)

Supplemental File 2 Cerebral Ultrasound initially (left) and at discharge (right) of six infants with intriguingly good course after intranasal breast milk. (JPG 1738 kb)

## Abbreviations

CPAP: Continuous positive airway pressure
cUS: Cerebral ultrasound
EGF: Epidermal growth factor
IVH: Intraventricular hemorrhage
MRI: Magnetic resonance imaging
NICU: Neonatal intensive care unit
PVL: Periventricular leukomalacia
RCT: Randomized controlled trial
VLBW: Very low birth weight
