# Supplementary figures and images for: Intranasal breast milk for premature infants with severe intraventricular hemorrhage—an observation
Source: Eur J Pediatr. 2018 Nov 1;178(2):199–206. doi: 10.1007/s00431-018-3279-7 (PMC6339661; doi:10.1007/s00431-018-3279-7)

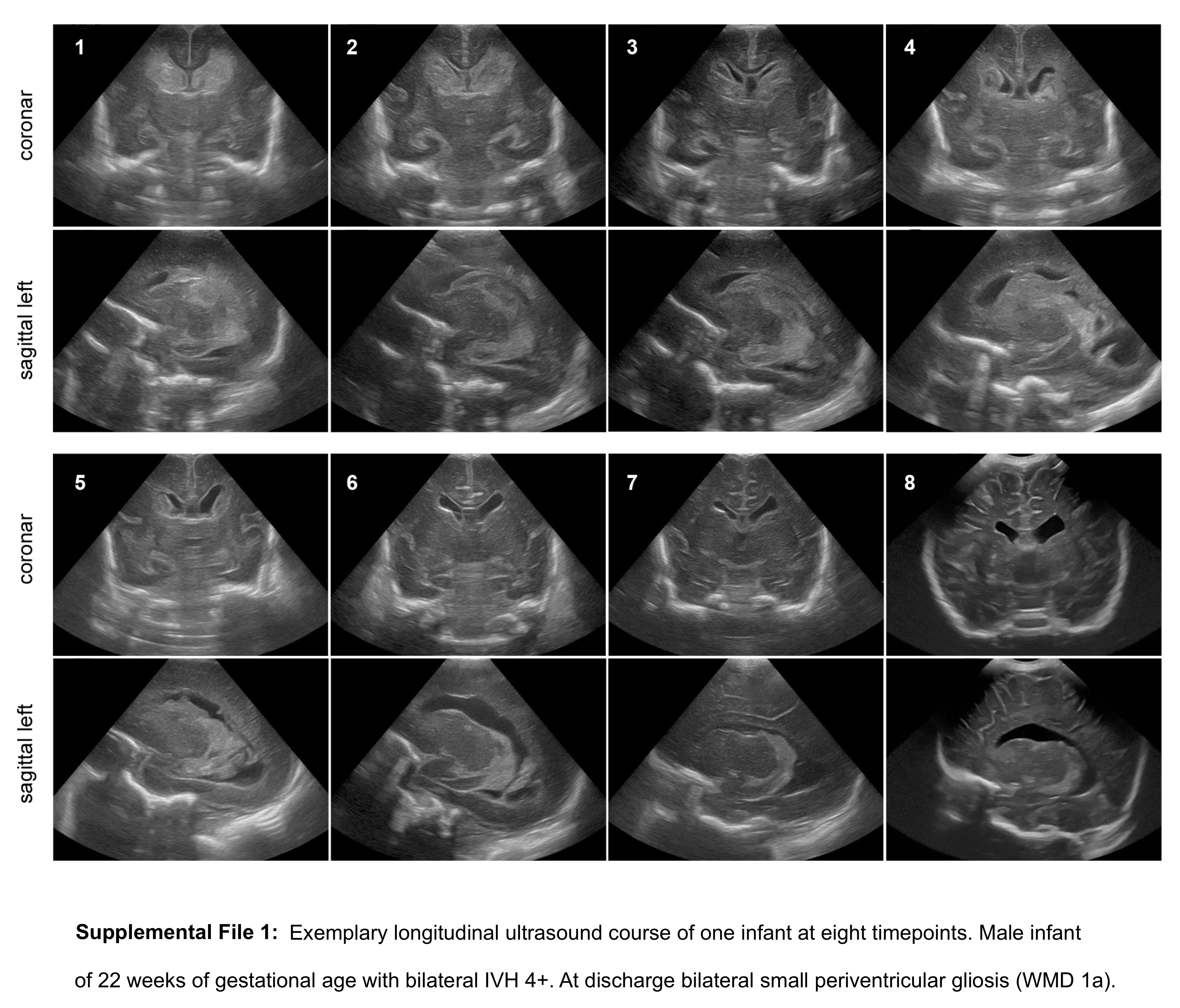

Supplement: Supplementary file 1 — Exemplary longitudinal cerebral ultrasound course of one infant at eight timepoints. Male infant of 22 weeks of gestational age with bilateral IVH 4+. At discharge bilateral small periventricular gliosis. (JPG 3496 kb) [file 431_2018_3279_MOESM1_ESM.jpg]

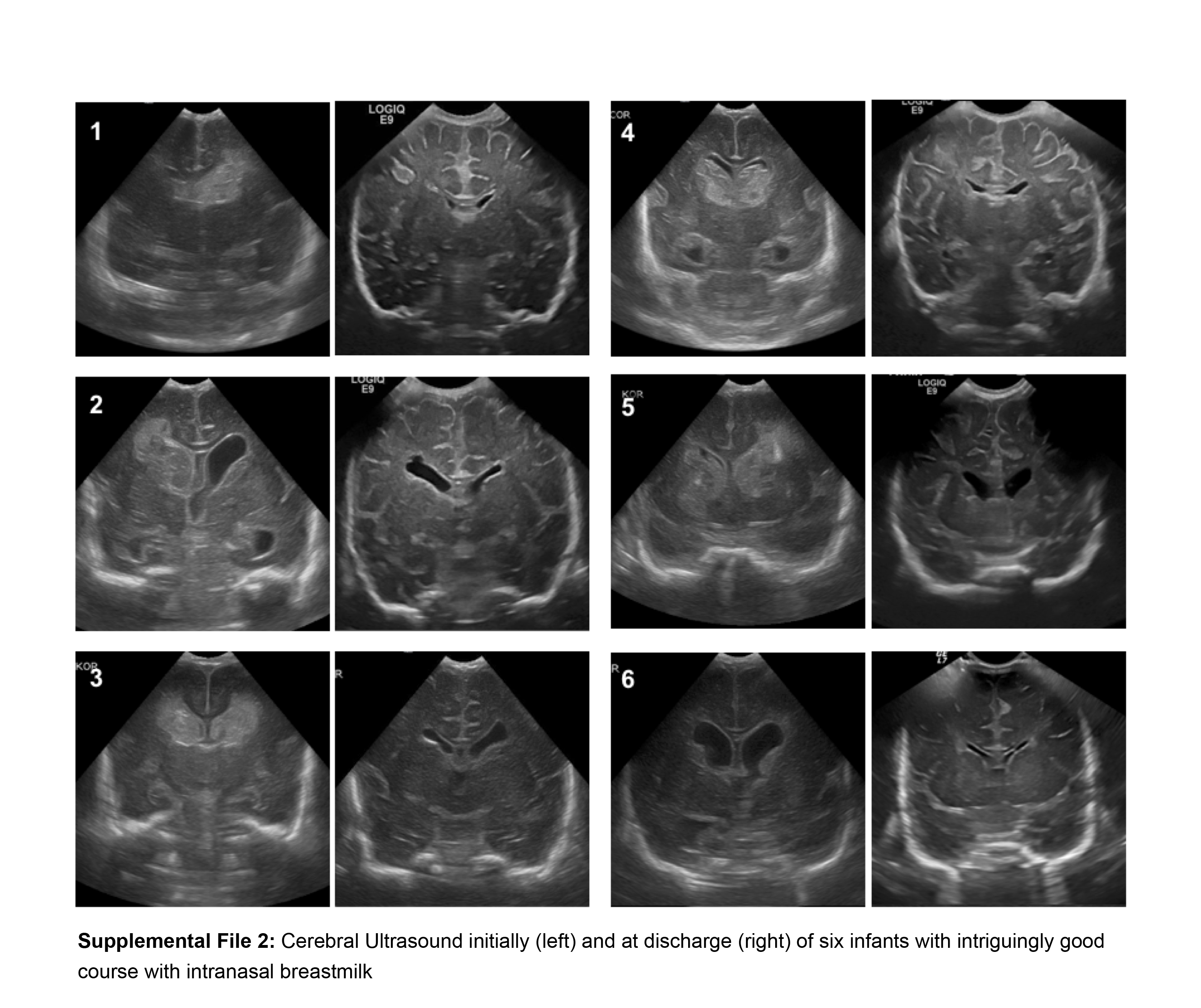

Supplement: Supplementary file 2 — Cerebral Ultrasound initially (left) and at discharge (right) of six infants with intriguingly good course after intranasal breast milk. (JPG 1738 kb) [file 431_2018_3279_MOESM2_ESM.jpg]
